# Guiding post-pancreaticoduodenectomy interventions for pancreatic cancer patients utilizing decision tree models

**DOI:** 10.3389/fonc.2024.1399297

**Published:** 2024-05-30

**Authors:** Haixin Wang, Bo Shen, Peiheng Jia, Hao Li, Xuemei Bai, Yaru Li, Kang Xu, Pengzhen Hu, Li Ding, Na Xu, Xiaoxiao Xia, Yong Fang, Hebing Chen, Yan Zhang, Shutong Yue

**Affiliations:** ^1^ Department of Cadre Medical, The First Medical Centre, Chinese People's Liberation Army (PLA) General Hospital, Beijing, China; ^2^ Department of Respiratory and Critical Care Medicine, The Eighth Medical Centre, Chinese People's Liberation Army (PLA) General Hospital, Beijing, China; ^3^ Academy of Military Medical Science, Beijing, China; ^4^ School of Software, Shandong University, Jinan, China; ^5^ Northwestern Polytechnical University School of Life Sciences, Xi'an, China; ^6^ College of Mathematics and Systems Science, Shandong University of Science and Technology, Qingdao, China

**Keywords:** pancreaticoduodenectomy, pancreatic cancer, complications, machine learning, survival analysis

## Abstract

**Background:**

Pancreatic ductal adenocarcinoma (PDAC) is frequently diagnosed in advanced stages, necessitating pancreaticoduodenectomy (PD) as a primary therapeutic approach. However, PD surgery can engender intricate complications. Thus, understanding the factors influencing postoperative complications documented in electronic medical records and their impact on survival rates is crucial for improving overall patient outcomes.

**Methods:**

A total of 749 patients were divided into two groups: 598 (79.84%) chose the RPD (Robotic pancreaticoduodenectomy) procedure and 151 (20.16%) chose the LPD (Laparoscopic pancreaticoduodenectomy) procedure. We used correlation analysis, survival analysis, and decision tree models to find the similarities and differences about postoperative complications and prognostic survival.

**Results:**

Pancreatic cancer, known for its aggressiveness, often requires pancreaticoduodenectomy as an effective treatment. In predictive models, both BMI and surgery duration weigh heavily. Lower BMI correlates with longer survival, while patients with heart disease and diabetes have lower survival rates. Complications like delayed gastric emptying, pancreatic fistula, and infection are closely linked post-surgery, prompting conjectures about their causal mechanisms. Interestingly, we found no significant correlation between nasogastric tube removal timing and delayed gastric emptying, suggesting its prompt removal post-decompression.

**Conclusion:**

This study aimed to explore predictive factors for postoperative complications and survival in PD patients. Effective predictive models enable early identification of high-risk individuals, allowing timely interventions. Higher BMI, heart disease, or diabetes significantly reduce survival rates in pancreatic cancer patients post-PD. Additionally, there’s no significant correlation between DGE incidence and postoperative extubation time, necessitating further investigation into its interaction with pancreatic fistula and infection.

## Introduction

Pancreatic cancer, characterized by uncontrolled cell growth in the pancreas, poses a significant health threat (1, 2). The 2023 projection report from the International Cancer Center anticipates 64,050 new cases and 50,550 deaths. Despite ranking 14th globally in incidence, it is the 7th leading cause of malignant tumour-related deaths. Alarmingly, the overall 5-year survival rate remains less than 5% (3). Surgical resection is the primary effective treatment for offering a chance of cure and prolonged survival (4–7). Extensive research has elucidated the safety and feasibility of laparoscopic and robotic pancreaticoduodenectomy, highlighting advantages such as minimized blood loss, reduced wound infection risk, and shortened hospital stays compared to open pancreaticoduodenectomy (OPD). However, this minimally invasive approach may entail prolonged operative durations (8, 9). While some intraoperative differences exist between laparoscopic pancreaticoduodenectomy (LPD) and OPD, current research suggests no appreciable disparities in mortality outcomes.

The perioperative mortality rate for pancreaticoduodenectomy (PD) has decreased to below 5% in major surgical centers with extensive experience (10). However, despite this low mortality rate, postoperative complications occur in up to 50% of cases, including surgical site infections, delayed gastric emptying (DGE), and pancreatic fistula (POPF) (11). Therefore, identifying intraoperative factors contributing to complications is paramount for optimizing postoperative recovery in PD patients.

Delayed gastric emptying (DGE) stands as the prevailing pancreas-specific complication (PSC) post-pancreaticoduodenectomy (PD), particularly prevalent among patients with pancreatic tumours who commonly experience malnutrition. To address nutritional needs and mitigate complications, clinicians frequently opt for nasogastric (NG) tube insertion alongside total parenteral nutrition (TPN) following PD. Despite the rationale behind prolonged NG tube placement post-pancreatic surgery, which aims to reduce complications such as pancreatic fistula and delayed gastric emptying, evidence from meta-analyses challenges its efficacy in reducing gastrointestinal anastomotic leakage (12). Conversely, prolonged nasogastric tube placement post-abdominal surgery is associated with a notable increase in post-pancreatic surgery pulmonary complications (PPCs) and delayed intestinal function recovery. Recommendations advise prompt removal of the nasogastric tube before emergence from anesthesia (13), and emerging articles question the necessity of routine nasogastric suction following pancreatic resection (14, 15). Consequently, controversy persists regarding the optimal timing for nasogastric tube removal in patients post-PD.

Due to the elevated incidence of complications following pancreaticoduodenectomy (PD) surgery, our objective is to leverage preoperative and intraoperative indicators. We employ a machine learning model to prognosticate each patient’s postoperative complications and survival trajectory subsequent to discharge. This predictive framework facilitates anticipating patients’ recovery status post-surgery and pre-discharge, enabling healthcare practitioners to enact targeted modifications in postoperative pharmaceutical interventions and diagnostic assessments.

In this retrospective investigation, we aggregated electronic health records of pancreatic cancer patients who underwent pancreaticoduodenectomy, delineating two binary decision tree models to forecast the likelihood of postoperative complications and one-year survival status following surgery. Subsequently, commencing from the model’s weighting, we delved into the correlation between pivotal factors influencing patients’ survival continuum and postoperative complications, proffering judicious recommendations for postoperative patient care grounded in clinical expertise.

## Materials and methods

### Study population

We enrolled 749 participants in a retrospective case-registration study who underwent pancreaticoduodenectomy for pancreatic cancer between July 2021 and February 2023. The research adhered to the Declarations of Helsinki and Istanbul and received approval from The First Medical Centre, Chinese PLA General Hospital institutional review committee (Ethical Approval No. S2021-134-01). Written consent was obtained from all participants. To ensure representation of the actual gender-age distribution, we included patients with complete medical records for subsequent analysis. Follow-up assessments were conducted annually via telephone interviews to ascertain patients’ recovery and survival status. Among the 459 patients with follow-up data, 74 were lost to follow-up. In cases of mortality, the family members of the deceased provided information regarding the time and cause of death.

Detailed covariate data, encompassing age, gender, nutrition score, and medical history (e.g., hypertension, diabetes, and myocardial infarction), were collected from admission evaluations. Tumor size was determined based on dimensions provided in pathology reports. Cancer staging followed standard classifications, categorizing tumours as localized (stage I), locally advanced (stage II), metastatic (stage III), or unknown. Drawing upon authoritative clinical guidelines and extensive clinical experience, we established criteria for defining postoperative complications as follows (16, 17):

(1) Surgical site infection (SSI) was diagnosed in patients exhibiting symptoms such as erythema, localized pain, persistent pyrexia, and wound dehiscence 3-7 days post-surgery, accompanied by abnormal levels of C-reactive protein (CRP) and white blood cell count (WBC) in laboratory tests.
(2) Delayed gastric emptying (DGE) was defined according to the criteria established by the International Study Group on Pancreatic Fistula (ISGPF), including the need for nasogastric tube placement for three days post-surgery, reinsertion of the nasogastric tube due to persistent vomiting, or an inability to tolerate solid food before the surgery date.
(3) Postoperative pancreatic fistula was assessed based on drainage fluid amylase levels exceeding three times the upper limit of normal on the third day or later post-surgery.

### Statistical analysis

We investigated the relationship between prediagnostic BMI and survival using Cox proportional hazards regression, deriving hazard ratios (HRs) and 95% confidence intervals (CIs) for mortality across BMI categories. Two sequential cohorts of patient samples were collected based on whether surgery occurred within one year from the date of sample collection (March 15, 2023). Due to the second cohort’s proximity to the time of analysis, precluding valid prognostic information retrieval through follow-up visits, only data from the initial cohort were utilized for subsequent survival analysis. Survival curves were constructed employing the Kaplan-Meier method, with statistical significance assessed via the log-rank test. Covariates deemed most prone to confounding, including age at diagnosis, sex, nutrition score, surgical approach, tumor dimensions, cancer stage, and diagnostic period, were incorporated into multivariable-adjusted models to account for potential biases over time.

Null values were excluded for a comparative analysis between laparoscopic pancreaticoduodenectomy (LPD) and robotic pancreaticoduodenectomy (RPD). Subsequently, nasogastric tube extubation and intraoperative bleeding time were dichotomized and quadratomized, respectively, with mean and variance calculated for each group to facilitate comparison.

To assess correlations between postoperative complications, ward.D2 clustering and h-clust ranking were utilized, employing Euclidean distance as the metric. Pearson, Spearman, and Kendall correlation coefficients were computed, with Spearman coefficients exhibiting greater sensitivity to group differences and fluctuations in positive and negative variables.

In our notation, “R” denotes the correlation coefficient, while “p” represents the statistical significance level used in hypothesis testing to determine the rejection of the null hypothesis. Statistical analyses were conducted using R version 4.2.2, with p-values derived from t-tests and chi-square tests. All p-values are two-sided, with significance defined as p < 0.05.

### Model architecture

The machine learning model developed for predicting a patient’s risk of death and risk of postoperative complications from their medical records comprises several key components: (1) Feature Selection and Processing: Various patient features, such as age, gender, surgical procedure, and surgery duration, are incorporated as inputs into the model. These features undergo screening and preprocessing to ensure a comprehensive and informative dataset for model training. (2) Data preprocessing: The data preprocessing process involves the handling of three types of data. Firstly, there are laparoscopic and robotic surgery data, perioperative outcome data, and patient feature data. Perioperative outcome data undergo column deletion, missing value handling, and data type conversion to ensure data quality. For the merging of laparoscopic and robotic surgery data, column selection and cleaning are conducted, and the data is merged using inner join. Finally, the data is divided into label and feature parts, and the features are normalized. Additionally, undersampling techniques are applied to balance the distribution of label categories, aiming to enhance the model’s generalization ability. (3) Decision Tree Model: The prediction of both the risk of death and the risk of postoperative complications is accomplished using a decision tree model. Due to the small sample size and simple features, we chose the decision tree model instead of the performance superior deep learning models on large-scale datasets. This model structure offers an intuitive representation of the relationships between input features and outcomes, facilitating easy interpretation for physicians. The decision tree delineates decision rules based on feature values, enabling a transparent and understandable basis for prediction. For the inference time of the two models, we conducted one hundred experiments and obtained an average of 0.99 × 10^-3^ seconds. This is almost imperceptible to doctors.

To prevent overfitting and assess the generalizability of the model predictions, we adopt a 9:1 ratio to randomly partition the dataset into training and test sets. The model is exclusively trained on the training set, and its performance is subsequently evaluated on the independent test set to ascertain its robustness and effectiveness.

In specific, the input indicators include: Operation time, Height, Age, Postoperative time to defecation, Nutrition score, BMI, EBL, Operation, Diabetes, Gastric tube extubation time, Postoperative time to flatus, Infection, POPF, Pancreatitis, Hypertension, DGE, Tumor volume, Operation, Body weight, Postoperative time to nasogastric tube removal, Intraoperative blood loss, Gender. These indicators are common measurements during patient surgeries. The output result is a binary number, either 0 or 1. In which, 0 denotes no postoperative mortality risk or complication risk, while 1 denotes postoperative mortality risk or complication risk. For medical professionals, inputting patient indicators into the model allows them to determine the presence of postoperative mortality and complication risks. This result can aid doctors in deciding whether intervention treatment is needed for the patient.

## Results

### Machine learning models for complication and survival status prognostication

**Figure 1:**
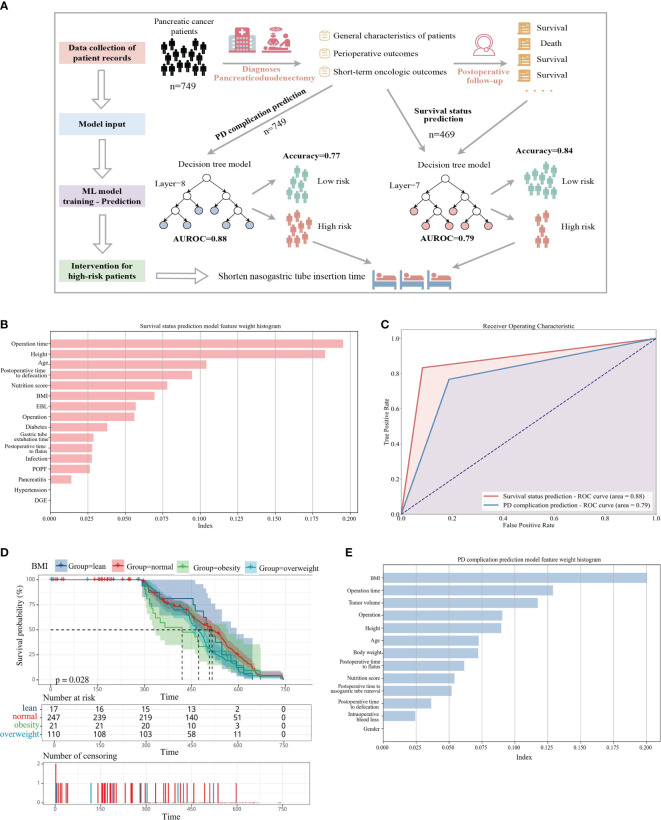
**(A)** Predicting the risk of complications and mortality from hospital electronic medical records. The workflow first collects the basic pathological characteristics and perioperative results of pancreatic cancer patients upon admission. The second step is to divide the training set and the test set according to 9:1 to build a complication prediction model. The third step is to collect the follow-up of patients one year after discharge. Data to build a survival state prediction model. Prompt postoperative intervention through risk assessment. **(B)** Assessment of prediction accuracy in high-risk patients. **(C)** The top 14 features that help predict survival status **(E)** The top 12 features that help predict PD surgical complications, all ranked according to contribution weight. **(D)** Kaplan-Meier curves of overall survival by baseline body mass index (BMI). Number at risk table: a table containing the number of surviving samples from each group at multiple time points. Number of censoring: table of the number of censored samples at each time point on the time axis for the four classes of groups classified by BMI.

Artificial intelligence (AI) techniques have been widely employed in numerous clinical decision support tasks, including cancer risk assessment (18–20). We conducted data extraction from patients’ general characteristics (**Supplementary Table S1**), perioperative outcomes (**Supplementary Table S2**), short-term oncologic outcomes (**Supplementary Table S3**), and discharge follow-up records, subsequently assessing various machine learning methodologies. Eventually, the decision tree model exhibiting the most favorable performance was identified, and a predictive model of high efficacy was constructed using a restricted training dataset (**Figure 1A**). Model algorithms possess the capability to deduce the influence of specific input variables on the predictive model (18). In the survival status prediction model, the model achieved a best Area Under the Receiver Operating Characteristic Curve (AUCROC) of 0.88, with an accuracy of 0.833, precision of 0.8, recall of 0.67, and F1-score of 0.727. We compared it to a published machine learning prediction model for pancreatic cancer and found that our proposed model performed best among models trained with the same order of magnitude of samples, with AUROC values only lower than the cancer risk model proposed by Placido et al (18). using clinical case studies constructed from 9 million patients (**Supplementary Table S4**).

Upon analyzing the factors influencing the model, we observed that operation time and height had a pronounced impact, likely associated with the complexity of surgical procedures dictated by patients’ health conditions. Additionally, common indicators were depicted in the contribution ranking (**Figure 1B**). Regarding the complication prediction model, the model yielded the best AUCROC of 0.79, with an accuracy of 0.79, precision of 0.85, recall of 0.77, and F1-score of 0.80 (**Figure 1C**). We discerned that Body Mass Index (BMI) exerted the most significant influence on model weights, aligning with the premise that elevated prediagnostic BMI correlates with diminished survival rates in pancreatic cancer patients (21). Moreover, model accuracy was impacted by operation time, tumor volume, surgical approach, and height (**Figure 1E**).

To delve into the genuine impact of operation time on the primary weight of the prediction model, we plotted violin distribution diagrams of operation time in LPD and RPD across various intraoperative blood loss and postoperative nasogastric tube removal durations, utilizing unpaired t-tests and unpaired Chi-Squared Kruskal-Wallis tests. To mitigate individual outliers, we categorized intraoperative blood loss into four levels (x∈ (0,50), x≈50, x≈100, x∈(100,200)). Since a majority of patients had their nasogastric tubes removed on the first (42.62%) and second (32%) postoperative days, patients were divided into two groups (x∈ (0,1), x>1). The findings revealed significant differences in operation time corresponding to varying degrees of bleeding during similar surgical procedures, with a progressive inclination toward prolonged operative durations as bleeding intensified. Additionally, the proportion of high blood loss patients (>50 ml) was notably higher in LPD compared to RPD (LPD: 42.3%, 13.8%; RPD: 23.21%, 7.67%), and the median intraoperative blood loss in LPD exceeded that of RPD across all four groups. Examination of operation time in relation to nasogastric tube removal time revealed a positive correlation between longer tube indwelling durations and increased median operation time. Notably, within the same group, LPD exhibited higher median operation times than RPD. Several factors contribute to the elevated weight of operation time in predicting complications and survival status. Longer operation durations often signify more severe conditions and intricate suturing tasks, indicative of a more critical surgical process.

Furthermore, extended operation times may reduce patients’ chances of survival due to heightened bleeding volumes and enhanced wound recovery difficulty. Prolonged operation durations also coincide with lengthier nasogastric tube insertion times, which may potentially heighten the risk of postoperative infections (see **Supplementary Figure S4**). In conclusion, under appropriate circumstances, we advocate for RPD surgery due to its minimally invasive nature and reduced blood loss requirements. Postoperatively, enhanced exploration and vigilance regarding nasogastric tube insertion times are recommended to validate the correlation between extraction time and complications, thereby facilitating improved patient care.

### Factors influencing prognostic outcomes and survival

**Figure 2:**
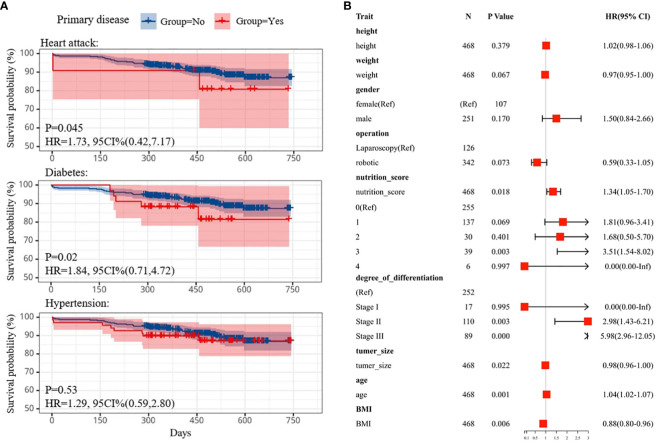
**(A)** Kaplan-Meier survival curve comparing survival of individuals with heart attack (top), diabetes(middle) and hypertension (bottom) subtypes. **(B)** Cox multifactor regression models were used to determine good and poor prognostic factors in patient attributes by calculating HR values. HR, hazard ratios.

Pancreatic cancer ranks as the fourth leading cause of cancer-related mortality in the United States (22). Among patients diagnosed with pancreatic adenocarcinoma, a mere 5% survive beyond 5 years, with the majority succumbing within 12 months of diagnosis. The complex interplay of various factors influences patient survival, yet reliable prognostic markers remain scarce. Utilizing a decision tree model to predict survival among pancreatic cancer patients, we conducted classical statistical survival analysis to identify key prognostic factors (see **Figure 2**).

Employing Kaplan-Meier curves, we assessed the impact of preoperative comorbidities, including hypertension, heart disease, and diabetes, on patient outcomes. Telephone follow-up one year post-surgery tracked the survival status of 469 patients. Notably, patients with preexisting heart disease or diabetes exhibited poorer prognoses compared to those without these conditions (p=0.045, HR=1.73, 95%CI (0.42,7.17); p=0.02, HR=1.84, 95%CI (0.71,4.72), respectively), as illustrated in **Figure 2A** (23). However, no statistically significant difference was observed in survival outcomes between hypertensive and normotensive patients (n = 156, p=0.53, Log-rank test).

Furthermore, we investigated the association between prediagnostic BMI and pancreatic cancer survival (see **Figure 1D**). Baseline characteristics of 749 pancreatic cancer patients were documented, revealing a mean age of 56.85 years and a mean baseline BMI of 23.76 kg/m2, with 27.9% categorized as overweight and 3.2% as obese. Kaplan-Meier analysis unveiled that higher baseline BMI correlated with reduced survival (p=0.028). Notably, the survival curves exhibited varying rates of decline across four subgroups, with lean patients displaying the slowest decline (n=34, median survival=510) and obese patients experiencing the swiftest decline (n=24, median survival=420). Among these subgroups, obese patients exhibited the shortest median survival (approximately 415 days), while those with normal BMI exhibited the longest (approximately 520 days), suggesting a plausible association between healthier preoperative BMI and prolonged survival.

Additionally, multivariate Cox analysis was employed to identify prognostic factors influencing pancreaticoduodenectomy (PD) outcomes (24) (see **Figure 2B**). Small BMI and tumor size were identified as favorable prognostic factors, positively associated with survival probability, whereas excessive nutritional status and tumor metastasis were deemed unfavorable prognostic factors. Lastly, survival probability curves for patients undergoing robotic pancreaticoduodenectomy (RPD) versus laparoscopic pancreaticoduodenectomy (LPD) were compared, revealing no significant difference in survival outcomes (see **Supplementary Figure S5**). The median survival time for both approaches was approximately 488 days [P=0.22, HR=1.16, 95% CI (0.91,1.49)], suggesting comparable efficacy. This aligns with existing literature indicating similar survival outcomes between the two procedures (9, 25–27).

In conclusion, while laparoscopic and robotic pancreaticoduodenectomy offer distinct advantages in bleeding control and operative time, respectively, their impact on patient survival appears equivalent. Enhanced outcomes for both procedures may ensue with increased surgical experience and refined patient selection.

### Putative correlates of postoperative complications

Given the high incidence of complications such as delayed gastric emptying (DGE), pancreatic fistula (POPF), and intra-abdominal infection post-pancreaticoduodenectomy (PD), coupled with the unclear causal mechanisms underlying these complications, we conducted a comprehensive analysis of 749 patient cases. Spearman correlation analysis, depicted in a heat map format (**Supplementary Figure S6**), revealed notable associations among complications, particularly highlighting robust correlations among DGE, POPF, and postoperative infection (correlation coefficients: DGE and postoperative infection, 0.33; POPF and postoperative infection, 0.18; POPF and postoperative infection, 0.26). We also explored the strength and significance of the association between complications by logistic regression analysis and Fisher’s exact test (**Supplementary Figures S7**, **S8**), and the results showed that when p-value < 0.01, there was a strong association between the three diseases, and the presence of one disease increased the risk of the other disease to varying degrees.

**Figure 3:**
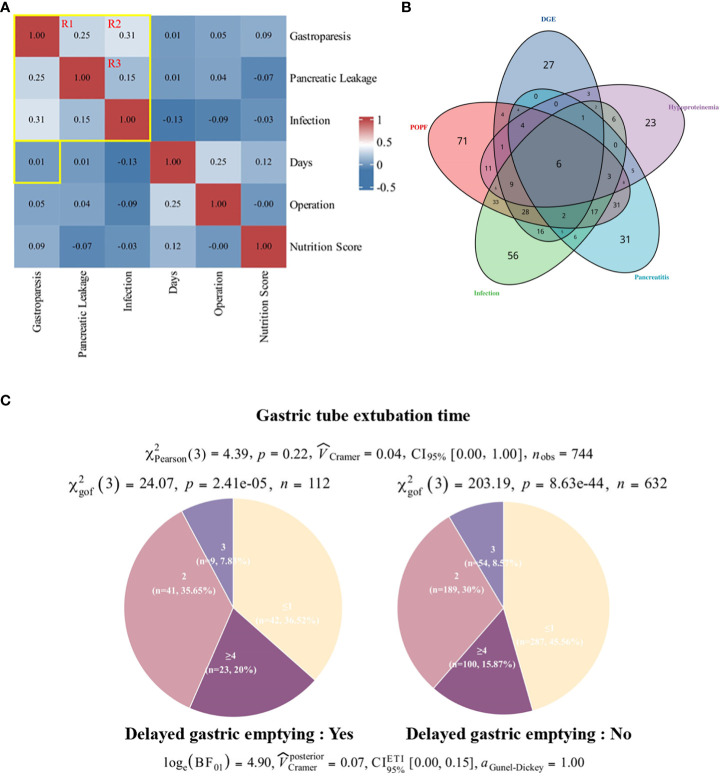
Correlation between incidence of DGE and time to gastric tube removal. **(A)** Patient postoperative large sample correlation heat map. Pearson correlation showed no significant correlation between DGE and days to extubation. there was a stronger association between DGE, POPF, and wound infection compared to other indicators. **(B)** Pie chart of the number of days to surgery for gastric tube removal in patients with DGE. **(C)** Overlap of complications. DGE, delayed gastric emptying; POPF, pancreatic fistula.

To further elucidate the interrelationship between major complications, we constructed Venn diagrams (see **Figure 3B**), revealing that over 80% of patients experienced multiple postoperative complications, potentially attributed to suboptimal lifestyle habits and compromised preoperative physical conditions. Notably, considerable overlap was observed among cases of DGE, POPF, and infection.

**Figure 4:**
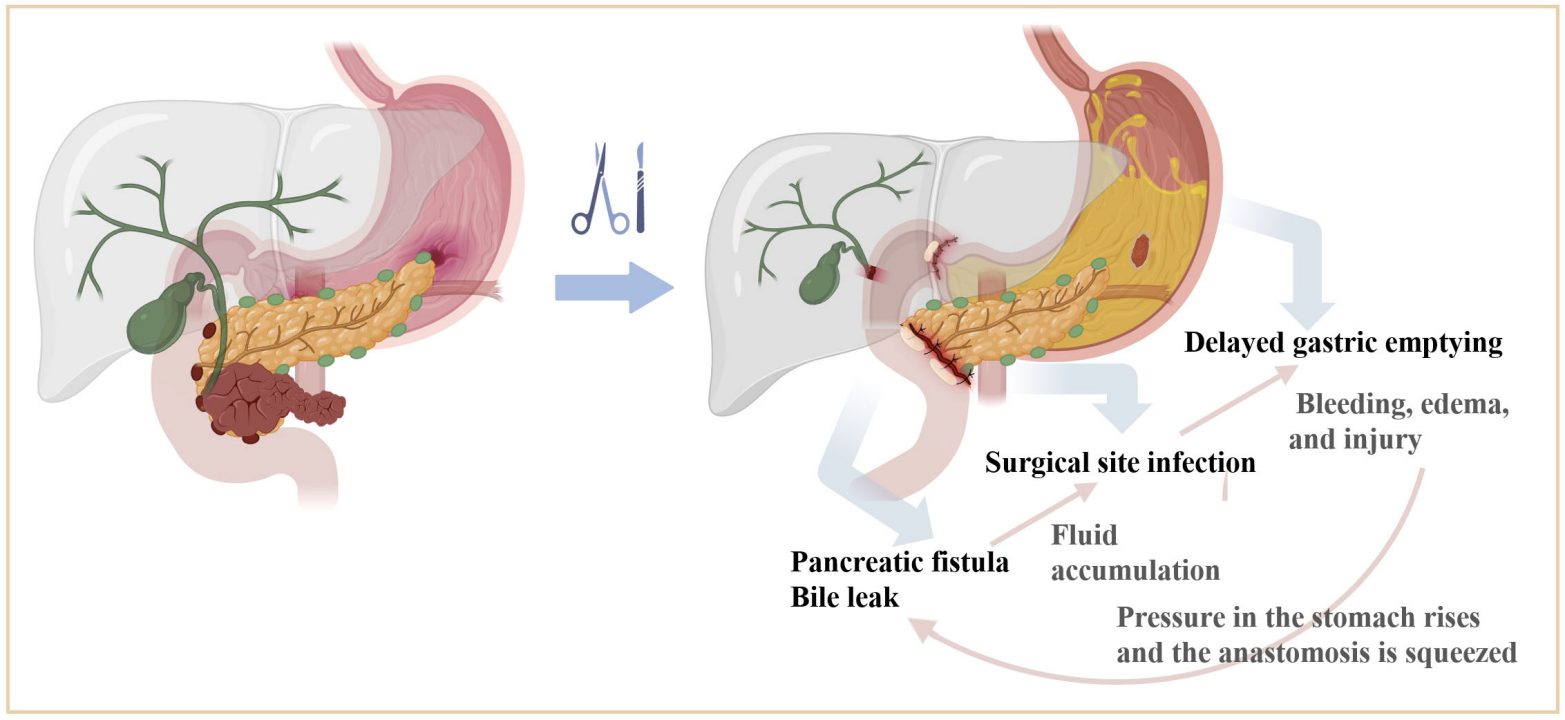
Mechanism of interaction between major complications after PD surgery. Pancreatobiliary leakage can cause secondary infection due to fluid accumulation in the surgical area, and a large number of inflammatory mediators accumulated in the surgical area can directly act on the surface of the gastrointestinal plasma membrane, causing bleeding, edema, and injury. Or indirectly inhibit gastrointestinal motility through the corresponding biologically active substances and bacterial endotoxins. Gastric paralysis will cause increased pressure in the stomach, so that the pancreatic and biliary anastomosis are squeezed, affecting the blood supply and healing of the anastomotic port, and further increasing the risk of anastomotic leakage or fistula formation. The three influence each other and worsen each other.

Considering the intricate interplay between these complications, the absence of definitive studies delineating their pathogenesis and causal nexus necessitates a cautious interpretation of their relationships, notwithstanding their high co-occurrence and modest correlations. Drawing from extensive clinical observations and a literature review, we propose a speculative causal mechanism linking the three major complications post-PD surgery (**Figure 4**).

Pancreatobiliary leakage may instigate secondary infections due to localized fluid accumulation, facilitating the release of inflammatory mediators that impede gastrointestinal function through edema, bleeding, and mucosal injury. Concurrently, gastric dysmotility can elevate gastric pressure, potentially compromising the integrity and perfusion of pancreatic jejunostomy and hepaticojejunostomy sites, thereby exacerbating the risk of anastomotic complications. The intricate interplay among these complications perpetuates a cycle of exacerbation, contributing to postoperative morbidity.

### Reducing the duration of nasogastric tube insertion facilitates postoperative recuperation

Primarily, the decision tree model indicates a noteworthy association between nasogastric tube insertion duration and patient prognosis in the survival prediction model. Concurrently, prolonged operative durations correlate with delayed extubation. Subsequently, based on extensive clinical observation, it is posited that early removal of the nasogastric tube, specifically on the initial postoperative day, not only mitigates patient discomfort but also facilitates expeditious initiation of oral intake, potentially enhancing patient well-being. Thus, from a postoperative care perspective, shortening the duration of nasogastric tube placement may expedite patient recovery.

To investigate the potential impact of nasogastric tube removal timing on Delayed Gastric Emptying (DGE), we conducted a comprehensive analysis employing a large-scale correlation heat map (see **Figure 3A**). Employing Spearman correlation coefficients, we evaluated pairwise relationships between variables, revealing no statistically significant association between postoperative nasogastric tube removal and DGE incidence (p=0.01) (see **Figure 3C**). Furthermore, to ascertain the independence of DGE prevalence from nasogastric tube extubation timing, we stratified the total sample into four subgroups (x∈ [0,1]; x=2; x=3; x∈ [4,23]) and performed chi-square tests within and across groups. Our analyses yielded statistically significant differences (sick: χ²_gof_(3)=24.07, p=2.41e^-5^; control: χ²_gof_(3)=203.19, p=8.63e^-44^) in the distribution of patients based on nasogastric tube removal timing. Interestingly, while the distribution patterns between the sick and control cohorts exhibited similarity, there was no statistically significant disparity (χ²_Pearson_(3)=4.39, p=0.22) between the two groups.

In conclusion, expeditious removal of the nasogastric tube upon the patient’s initial passage of flatus holds potential to alleviate discomfort, hasten oral intake adaptation, and potentially mitigate infection risk, thereby enhancing overall patient well-being.

## Conclusion

Over the past fifteen years, both laparoscopic pancreaticoduodenectomy (LPD) and robotic pancreaticoduodenectomy (RPD) have emerged as viable options for a spectrum of hepatic-pancreaticobiliary (HPB) procedures. Assessing the safety and feasibility of these surgical approaches is paramount (28, 29). This study embarked on an exhaustive retrospective case review involving 749 patients who underwent pancreaticoduodenectomy within the preceding two years, stratifying them into distinct cohorts based on their surgical categorization. We utilize preoperative clinical assessments and perioperative outcomes to construct two decision tree models to prognosticate the likelihood of postoperative complications and the survival status of patients one year post-surgery. Subsequent to identifying potential interrelationships among various complications post pancreaticoduodenectomy, we proposed a multifaceted causal mechanism delineating the mutual exacerbation between delayed gastric emptying (DGE), postoperative pancreatic fistula (POPF), postoperative infection, and the into digestive system milieu perturbed by neoplastic lesions. However, given the complexity of this dynamic, prospective randomized controlled trials are warranted to elucidate the underlying pathophysiological pathways comprehensively.

In a prognostic examination of pancreaticoduodenectomy patients, our investigation revealed that preoperative diagnoses of heart disease and diabetes were associated with diminished postoperative survival rates (30). Within this expansive retrospective inquiry incorporating Body Mass Index (BMI) data, a negative correlation between preoperative BMI and survival among pancreatic cancer patients was discerned, particularly noting adverse survival outcomes among obese individuals in the years leading up to diagnosis. Remarkably, the median survival duration within this patient cohort approximated 16 months, substantially surpassing the reference median survival period of 4.4 months reported by the National Cancer Center. Given that the median age of the cohort patients stood at 59 years, significantly lower than the reference age of 70 years, it is plausible that, alongside the advanced healthcare infrastructure and proficient medical personnel in the hospital setting, the relatively younger age of the patients, coupled with early cancer detection and timely interventions, contributed significantly to prolonged survival.

Furthermore, elevated nutritional ratings, low-to-moderate tumor differentiation, and advanced age were identified as adverse prognostic indicators for patients. These findings underscore the impact of chronic systemic metabolic alterations on pancreatic cancer patient survival, emphasizing the deleterious consequences of underlying comorbidities resulting from unhealthy lifestyle habits on patient recovery. Moreover, no significant discrepancy in survival outcomes exists between the LPD and RPD approaches, enabling patients to make informed choices regarding the most suitable surgical modality based on other pertinent considerations such as operative duration, hemorrhage, and hospitalization duration.

Significantly, our analysis revealed no association between delayed gastric emptying (DGE) incidence and the duration from pancreaticoduodenal surgery to nasogastric tube removal, as evidenced by the distribution of nasogastric tube removal times. Additionally, considering the impact of nasogastric tube removal on the survival model, we advocate for the prompt removal of the nasogastric tube following the occurrence of the patient’s first flatus during the postoperative care phase.

Postoperative complications following pancreaticoduodenectomy (PD) represent critical risk factors influencing patient prognosis, with delayed gastric emptying (DGE), postoperative pancreatic fistula (POPF), infection, and postoperative pancreatitis being the most prevalent. Notably, a modest correlation was observed between DGE, POPF, and infection compared to other variables in the correlation heat map. Furthermore, our analysis did not identify significant correlations between preoperative comorbidities and postoperative complications, potentially attributable to sample size limitations. Hence, future studies necessitate expanded sample sizes and prolonged follow-up periods to facilitate more comprehensive analytical investigations.

Existing literature predominantly focuses on individual complications, overlooking the pathogenesis of each ailment and the interplay among the three complications. In this context, we posit that the underlying mechanism linking these complications may involve the accumulation of inflammatory mediators within the surgical region, exerting direct effects on the gastrointestinal epithelium and inducing bleeding, edema, and injury. Pancreatobiliary leakage may precipitate secondary infections due to fluid accumulation, while the presence of physiologically active substances and bacterial endotoxins may impede gastrointestinal motility. Furthermore, gastric paralysis resulting from these complications may constrict pancreatic and biliary anastomoses, elevating the risk of leakage or fistula formation and impeding vascular perfusion and healing at the anastomotic site.

In mitigating postoperative complications arising from surgical procedures, the literature suggests several strategies, including the use of wound protectors to reduce the risk of surgical site infections, minimizing intraoperative bleeding, and employing fistula tubes larger than 5mm to mitigate the incidence of pancreatic fistula (31–33). Additionally, techniques such as colon anterior resection and preoperative biliary drainage have shown promise in reducing DGE rates (34). Adequate preoperative antibiotic prophylaxis can also diminish the incidence rates of surgical site infections, POPF, and Clostridium infections (35, 36). Given the diverse array of postoperative complications associated with PD, substantial efforts are warranted to explore and implement novel therapeutic modalities for pancreatic cancer patients (37).

## Data availability statement

The datasets presented in this article are not readily available because the data used in this back-looking study relates to the clinical history of the patient at the time of the visit, and we are obliged to protect the privacy of the patient. Requests to access the datasets should be directed to SY, ShuTongyue1999@gmail.com.

## Ethics statement

The studies involving humans were approved by The First Medical Centre, Chinese PLA General Hospital institutional review committee (Ethical Approval No. S2021-134-01). The studies were conducted in accordance with the local legislation and institutional requirements. The participants provided their written informed consent to participate in this study.

## Author contributions

SY: Conceptualization, Data curation, Formal analysis, Investigation, Methodology, Project administration, Software, Validation, Visualization, Writing – original draft, Writing – review & editing. YC: Conceptualization, Resources, Validation, Writing – review & editing. PJ: Conceptualization, Software, Writing – review & editing. HL: Funding acquisition, Investigation, Supervision, Validation, Writing – review & editing. XB: Investigation, Project administration, Writing – original draft. YL: Conceptualization, Data curation, Project administration, Visualization, Writing – original draft. KX: Formal analysis, Software, Validation, Writing – review & editing. BS: Investigation, Supervision, Writing – original draft. PH: Resources, Software, Writing – original draft. LD: Investigation, Writing – original draft. XX: Investigation, Validation, Writing – original draft. YZ: Investigation, Methodology, Resources, Supervision, Validation, Writing – original draft. YF: Data curation, Formal analysis, Project administration, Software, Supervision, Visualization, Writing – review & editing. HW: Data curation, Investigation, Resources, Validation, Visualization, Writing – review & editing. HC: Conceptualization, Funding acquisition, Supervision, Writing – review & editing. NX: Investigation, Writing – original draft.
