# Supplementary material for: Guiding post-pancreaticoduodenectomy interventions for pancreatic cancer patients utilizing decision tree models
Source: Front Oncol. 2024 May 30;14:1399297. doi: 10.3389/fonc.2024.1399297 (PMC11169653; doi:10.3389/fonc.2024.1399297)
Supplement: Supplementary file 1 [file DataSheet_1.docx]

Supplementary Material

# SupplementaryFigures


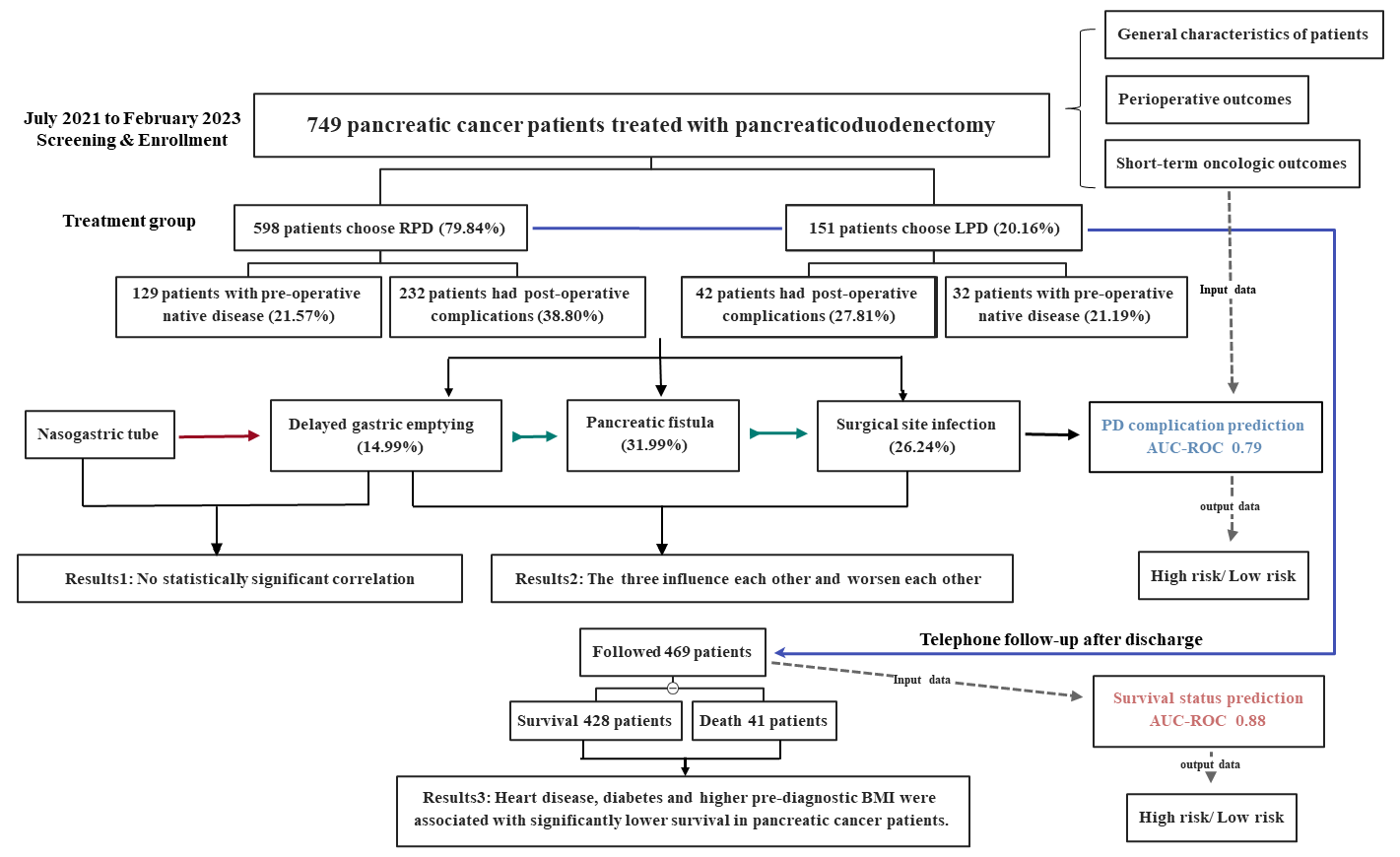


**Supplementary Figure 1.** A flow chart for surgical options and preoperative & postoperative disease in pancreatic cancer patients. LPD, laparoscopic pancreaticoduodenectomies; RPD, robotic pancreaticoduodenectomies; pre-operative native disease include patients which was admitted to the hospital for examination of hypertension, diabetic and heart disease. post-operative complications include delayed gastric emptying, pancreatic fistula, wound infection hypoproteinemia, pancreatitis, bile leak and chyle fistula. After the patients were discharged, we conducted telephone follow-ups on 469 patients and conducted PD survival analyses using the patients' survival status. We collected and summarized the results of patients’ preoperative physical examination, intraoperative perioperative report and postoperative recovery follow-up and built a machine learning model to predict complications and survival status


**Supplementary Figure 2.** CONSORT Flow Diagram. We recruited subjects from 749 participants of the retrospective case-registration study, who had undergone pancreaticoduodenectomy for Pancreatic cancer from july2021 to February 2023. To reflect the true patient gender-age distribution, all patients with complete medical records were selected for subsequent analysis in this paper. We updated patients' recovery and survival status by annual telephone follow-up, Of the 459 patients with follow-up records, 74 were lost and in the case of death, the family of the deceased provided the time and cause of death. Detailed covariate data, including age, gender, nutrition score and History of underlying disease(hypertension、diabetes and heart attack), were obtained from admission examination.

**Supplementary Figure 3.** Comparison of perioperative outcomes between LPD and RPD. a) Distribution of intraoperative bleeding and operative time by type of operation. b) Distribution of days to surgery for gastric tube removal versus surgery time by type of surgery.

**Supplementary Figure 4.** The correlation between some key patient indicators and postoperative complications was described using the Pearson correlation index. The results showed that whether the tumor is an endocrine tumor or an exocrine tumor has no significant impact on postoperative complications; since endocrine tumors are sporadic, accounting for only 4.8% of the sample, we reasonably speculate that the reason for this weak correlation may be due to the smaller sample size Smaller weights in correlation analyzes prevent data points in smaller samples from being given the weight they deserve in larger samples. Compared with other factors, we found that the time to nasogastric tube removal was associated with postoperative infection (p=-0.14). The sooner the nasogastric tube is removed, the less likely the patient is to develop postoperative infection.

**Supplementary Figure 5.** Kaplan-Meier survival curve comparing survival of different operations. There was no significant difference in the probability of survival between patients with LPD surgery and RPD surgery.

**Supplementary Figure 6.** Spearman correlation heat map of common complications after PD surgery in patients with pancreatic cancer.


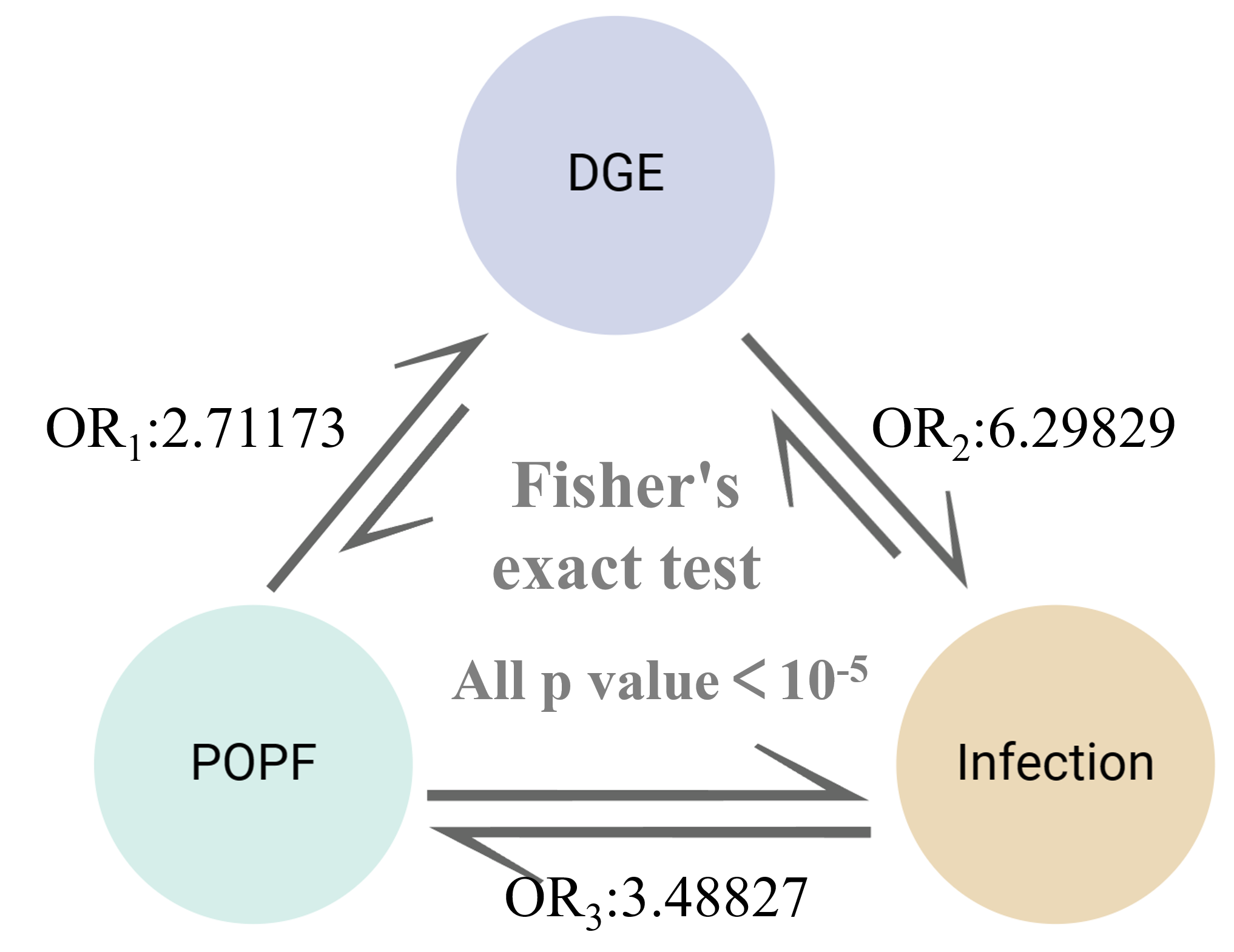


**Supplementary Figure 7.** The Fisher Precision Test explores the relationship between delayed gastric emptying, pancreatic fistula, and wound infection. We performed pairwise statistical tests for the three complications, and used the odds ratio (OR) to quantify the strength of the association between the two diseases. The results showed that there was a strong positive association between complications when the p-value was 10-5, indicating that the correlation between gastric paralysis and postoperative infection was the highest (OR2=6.29829, p≈0.000), followed by pancreatic fistula and postoperative infection (OR3=3.48827, p≈ 0.00), and finally gastric paralysis and pancreatic fistula, which were more regarded as independent variables (OR=2.71173, p≈0.000003).


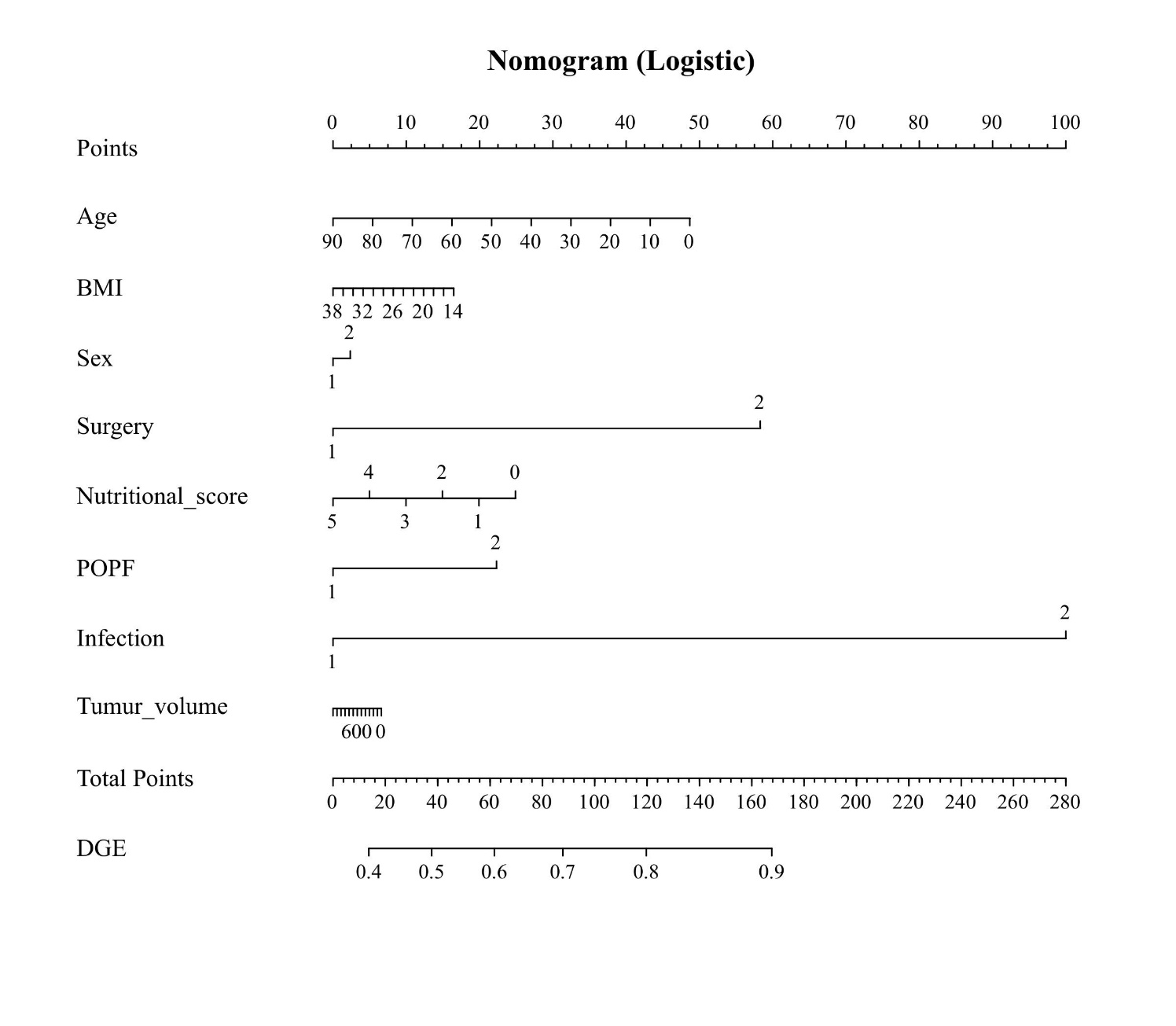


**Supplementary Figure 8.** Logistic regression model Nomo diagram. The model scores all the values of each variable, and the total score is used to quantify the probability that patients with pancreatic cancer will not suffer from delayed gastric emptying after surgery. As can be seen from the figure, no wound infection and pancreatic fistula occur after surgery, and a younger age, as well as a lower BMI and nutritional rating, will obtain a higher overall score, representing a lower risk of disease.
