# Supplementary material for: Guiding post-pancreaticoduodenectomy interventions for pancreatic cancer patients utilizing decision tree models
Source: Front Oncol. 2024 May 30;14:1399297. doi: 10.3389/fonc.2024.1399297 (PMC11169653; doi:10.3389/fonc.2024.1399297)
Supplement: Supplementary file 2 [file DataSheet_2.docx]

# TABLES

- **Table S1.** General characteristics of patients

| **Characteristics** | **RPD(N=598)** | **LPD(N=151)** | **Total(N=749)** | **pvalue** | **FDR** |
| --- | --- | --- | --- | --- | --- |
| Age |  |  |  |  |  |
| Mean±SD | 56.99±14.33 | 56.26±13.24 | 56.85±14.11 |  |  |
| Median[min-max] | 59.00[9.00,86.00] | 58.00[19.00,81.00] |  |  |  |
| BMI(kg/m^2^) |  |  |  |  |  |
| Mean±SD | 23.77±3.25 | 23.72±3.55 | 23.76±3.31 |  |  |
| Median[min-max] | 23.36[14.53,36.52] | 23.15[15.12,33.97] | 23.36[14.53,36.52] |  |  |
| Gender |  |  |  | 0.04 | 0.26 |
| Male | 302(40.37%) | 90(12.03%) | 392(52.41%) |  |  |
| Female | 296(39.57%) | 60(8.02%) | 356(47.59%) |  |  |
| Nutrition score |  |  |  | 0.44 | 1 |
| 5 | 1(0.13%) | 0 | 1(0.13%) |  |  |
| 4 | 10(1.34%) | 1(0.13%) | 11(1.47%) |  |  |
| 3 | 48(6.41%) | 18(2.40%) | 66(8.81%) |  |  |
| 2 | 47(6.28%) | 7(0.93%) | 54(7.21%) |  |  |
| 1 | 171(22.83%) | 41(5.47%) | 212(28.30%) |  |  |
| 0 | 321(42.86%) | 84(11.21%) | 405(54.07%) |  |  |
| Hypertension |  |  |  | 0.18 | 0.89 |
| Yes | 131(17.49%) | 25(3.34%) | 156(20.83%) |  |  |
| No | 467(62.35%) | 126(16.82%) | 593(79.17%) |  |  |
| Diabetes |  |  |  | 0.22 | 0.89 |
| Yes | 79(10.55%) | 14(1.87%) | 93(2.42%) |  |  |
| No | 519(69.29%) | 137(18.29%) | 656(87.58%) |  |  |
| Heart attack |  |  |  | 0.52 | 1 |
| Yes | 29(3.87%) | 5(0.67%) | 34(4.54%) |  |  |
| No | 569(75.97%) | 146(19.49%) | 715(95.46%) |  |  |
| Anemia |  |  |  | 1 | 1 |
| Yes | 3(0.40%) | 0 | 3(0.40%) |  |  |
| No | 595(79.44%) | 151(20.16%) | 746(99.60%) |  |  |

Baseline characteristics of the 749 included patients are summarized in Table1. The mean age of patients undergoing surgery at baseline was 56 years, and the median baseline BMI was 23.36kg/m2. Among the above-mentioned patients who underwent PD surgery, 21.50% had comorbidities and 36.58% had postoperative complications. There were no significant differences in age, BMI, nutritional score, and other preoperative plateau morbidity rates between the groups.

Abbreviations: FDR, false discovery rate; BMI, body mass index; RPD, robotic pancreaticoduodenectomy; LPD, laparoscopic pancreaticoduodenectomy.

**Table S2.** Perioperative outcomes

| **Characterists** | | **RPD(N=596)** | **LPD(N=151)** | **Total(N=747)** | **pvale** | **FDR** |
| --- | --- | --- | --- | --- | --- | --- |
| Operation time |  | |  |  |  |  |
| Mean±SD | 3.99±1.51 | | 5.11±2.05 | 4.22±1.69 |  |  |
| Median  [min-max] | 3.92[0.83,12.75] | | 5.02[1.08,11.83] | 4.00[0.83,12.75] |  |  |
| EBL |  | |  |  |  |  |
| Mean±SD | 91.77±130.95 | | 130.10±151.57 | 99.52±136.13 |  |  |
| Median  [min-max] | 50.00[5.00,1500.00] | | 100.00[5,1000] | 50.00[5.00,1500.00] |  |  |
| Time to first flatus |  | |  |  |  |  |
| Mean±SD | 3.30±2.23 | | 3.63±2.19 | 3.36±2.22 |  |  |
| Median  [min-max] | 3.00[1.00,22.00] | | 3.00[1.00,14.00] | 3.00[1.00,22.00] |  |  |
| Time to first defecation |  | |  |  |  |  |
| Mean±SD | 3.59±2.08 | | 3.69±2.50 | 3.61±2.17 |  |  |
| Median  [min-max] | 3.00[0,22.00] | | 3.00[0,14.00] | 3.00[0,22.00] |  |  |
| Gastric tube extubation time |  | |  |  |  |  |
| Mean±SD | 2.17±2.42 | | 3.08±2.32 | 2.35±2.43 |  |  |
| Median  [min-max] | 2.00[0,30.00] | | 2.00[0,14.00] | 2.00[0,30.00] |  |  |
| Delayed gastric emptying |  | |  |  | 9.20E-06 | 1.80E-05 |
| None | 3(0.40%) | | 6(0.80%) | 9(1.20%) |  |  |
| No | 490(65.60%) | | 136(18.21%) | 626(83.80%) |  |  |
| Yes | 103(13.79%) | | 9(1.20%) | 112(14.99%) |  |  |
| Pancreatic fistula |  | |  |  | 4.70E-12 | 1.40E-11 |
| None | 2(0.27%) | | 4(0.54%) | 6(0.80%) |  |  |
| No | 371(49.67%) | | 131(17.54%) | 502(67.20%) |  |  |
| Yes | 223(29.85%) | | 16(2.14%) | 239(31.99%) |  |  |
| Wound infection |  | |  |  | 8.90E-03 | 8.90E-03 |
| None | 3(0.40%) | | 5(0.67%) | 8(1.07%) |  |  |
| No | 430(57.56%) | | 113(15.13%) | 543(72.69%) |  |  |
| Yes | 163(21.82%) | | 33(4.42%) | 196(26.24%) |  |  |
| CRP |  | |  |  |  |  |
| Mean±SD | 7.08±4.86 | | 7.24±4.58 | 7.11±4.80 |  |  |
| Median  [min-max] | 6.43[0.16,24.80] | | 6.96[0.98,17.99] | 6.67[0.16,24.80] |  |  |
| WBC |  | |  |  |  |  |
| Mean±SD | 13.02±5.12 | | 12.79±3.97 | 12.98±4.94 |  |  |
| Median  [min-max] | 12.07[2.80,40.36] | | 12.23[6.50,24.44] | 12.10[2.80,40.36] |  |  |
| Bile leak |  | |  |  | 0.75 | 1 |
| Yes | 13(1.74%) | | 2(0.27%) | 15(2.01%) |  |  |
| No | 583(78.05%) | | 149(19.95%) | 732(97.99%) |  |  |
| Chyle fistula |  | |  |  | 0.59 | 1 |
| Yes | 5(0.67%) | | 0 | 5(0.67%) |  |  |
| No | 591(79.12%) | | 151(20.21%) | 742(99.33%) |  |  |
| Pancreatitis |  | |  |  | 3.00E-03 | 0.02 |
| Yes | 110(14.73%) | | 13(1.74%) | 123(16.47%) |  |  |
| No | 486(65.06%) | | 138(18.47%) | 624(83.53%) |  |  |
| Hypoproteinea |  | |  |  | 1.70E-05 | 9.90E-05 |
| Yes | 84(11.24%) | | 4(0.54%) | 88(11.78%) |  |  |
| No | 512(68.54%) | | 147(19.68%) | 659(88.22%) |  |  |
| Postoperative bleeding |  | |  |  | 0.28 | 0.84 |
| Yes | 20(2.68%) | | 2(0.27%) | 22(2.95%) |  |  |
| No | 576(77.11%) | | 149(19.95%) | 725(97.05%) |  |  |

The perioperative outcomes are summarized in Table2. In all rank variables, the proportion of postoperative complications of DGE (RPD17.3%; LPD 6.0%), POPF (RPD 37.4%; LPD 10.6%), wound infection (RPD 27.3%; LPD 21.9%), pancreatitis (RPD 18.5%; LPD 8.6%) and hypoproteinemia (RPD 14.1%; LPD 2.6%) was significantly higher in the RPD group than in the LPD group (P<0.01; pancreatitis FDR=0.02, others FDR<0.01). Abbreviations: FDR, false discovery rate. CRP, C-reactive protein; WBC, white blood cell count; EBL, estimated blood loss.

**TableS3** Short-term oncologic outcomes

| **Characteristics** | **RPD(N=598)** | **LPD(N=151)** | **Total(N=749)** | **pvalue** | **FDR** |
| --- | --- | --- | --- | --- | --- |
| Tumour size |  |  |  |  |  |
| Mean±SD | 34.93±90.16 | 40.91±98.51 | 36.13±91.86 |  |  |
| Median[min-max] | 10.00[0.03,1155.00] | 12.75[0.04,654.93] | 10.20[0.03,1155.00] |  |  |
| Degree of differentiation |  |  |  | 0.15 | 0.19 |
| Stage III | 126(33.33%) | 38(10.05%) | 164(43.39%) |  |  |
| Stage II | 145(38.36%) | 35(9.26%) | 180(47.62%) |  |  |
| Stage I | 31(8.20%) | 3(0.79%) | 34(8.99%) |  |  |
| Tumour types |  |  |  | 0.09 | 0.19 |
| Exocrine | 565(75.43%) | 148(19.76%) | 713(95.19%) |  |  |
| Endocrine | 33(4.41%) | 3(0.40%) | 36(4.81%) |  |  |
| PNI |  |  |  | 0.06 | 0.19 |
| None | 106(14.15%) | 18(2.40%) | 124(16.56%) |  |  |
| No | 391(52.20%) | 114(15.22%) | 505(67.42%) |  |  |
| Yes | 101(13.48%) | 19(2.54%) | 120(16.02%) |  |  |
|  |  |  |  |  |  |

Table3 shows the Short-term oncologic outcomes. Among those pancreatic cancer stage, 9.00% had highly differentiated (stage I) tumors, 47.62% had moderately differentiated (stage II) tumors, and 43.39% had hypo fractionated (stage III) tumors, which means cancer has spread to other organs. By tumor pathological category, 95.19% were exocrine tumors and 4.81% were endocrine tumors. The LPD group showed comparable short-term oncologic outcomes to those of RPD. No significant difference was found in the tumor size, degree of differentiation, tumor types and perineural invasion (PNI). Abbreviations: PNI, perineural invasion; FDR, false discovery rate.
